# A Clear-Sighted Focus On Climate- Friendly Operations Will Enhance Sustainable Marine Aquaculture Approaches

**DOI:** 10.1093/biosci/biac067

**Published:** 2022-08-26

**Authors:** Alice R Jones, Heidi K Alleway, Dominic McAfee, Patrick Reis-Santos, Seth J Theuerkauf, Robert C Jones

**Affiliations:** The University of Adelaide, Adelaide, South Australia, Australia; The Nature Conservancy, Arlington, Virginia, United States; The University of Adelaide, Adelaide, South Australia, Australia; The University of Adelaide, Adelaide, South Australia, Australia; NOAA National Marine Fisheries Service, Silver Spring, Maryland, United States; The Nature Conservancy, Arlington, Virginia, United States

In their response to our article (Jones et al. 2022), Jia and Chen (2022) suggest that our guidance for mitigating greenhouse gas (GHG) emissions from mariculture may result in “enormous environmental risks.” Their main criticism is that we omitted impacts from non-GHG-related pollution, such as excess nutrients and other “negative impacts on the whole system.” We agree that aquaculture has environmental sustainability challenges, which have been comprehensively researched and reported (e.g., Naylor et al. 2021). These environmental impacts are also the focus of international efforts by the United Nations, nongovernmental organizations, and third-party certification schemes. However, explicitly addressing mariculture's GHG footprint has largely been overlooked in such efforts. This motivated our specific focus on GHG emissions, an escalating challenge in food systems that must be tackled with a dedicated, clear-sighted focus.

Jia and Chen (2022) state that our study underestimates GHG footprint by overlooking “invisible emissions.” This is true and something we acknowledged, including guidance on how this may be improved (see the “Thorough carbon accounting is critical” section). Furthermore, we dedicated entire sections to the rarely quantified “invisible” GHG emissions caused by environmental degradation (see the “Interactions with surrounding marine environments influence GHG emissions” section and examples in the supplemental material). We illustrated the importance of ecological interactions on GHG emissions in Jones et al. 2022, in both figure 1 and box 1 (focused on the ocean carbon cycle).

The authors of the letter also, mistakenly, interpret our article as advocating for unchecked, large-scale mariculture development. This is not the case, although we note that it *is* a rapidly growing food production industry (FAO 2020). There is an opportunity to positively influence the climate impacts of the growing industry by incorporating climate-friendly approaches, in both existing and new operations. Advocacy for climate-smart approaches is not—and must never become—*a replacement* for general sustainable operation, but an *addition* to it. We also note the authors’ apparent misperception that growth in mariculture production can only be achieved through areal expansion. Growth can also be achieved by using existing areas better, increasing efficiency, and implementing integrated solutions (Belton et al. 2020).

Jia and Chen furthermore, commented that we had not accounted for “socioeconomic and ecological resilience.” While we appreciate the importance of these factors, they were not the focus of our substantial Overview, because they are so expansive and significant as to be complex research fields in their own right. These topics should be addressed with the depth of consideration that they warrant, rather than being included as an afterthought. A cursory take on resilience, considering only the negative effects of mariculture on ecosystems, could readily lead to the conclusions Jia and Chen have made in their letter. However, a growing body of work describes the potential for specific mariculture practices to positively contribute to ecosystems, which could support increased ecological resilience (see Barrett et al. 2022 as one example).

Ultimately, we agree with Jia and Chen that our article should be expanded through further work toward enabling locally relevant, climate-friendly practices to be embedded into sustainable mariculture operations. We hope that our work initiates these discussions and provides a valuable foundation for ongoing research and action on this topic.

## Author Biographical

Alice R. Jones (alice.jones01@adelaide.edu.au), Dominic McAfee, and Patrick Reis-Santos are affiliated with the School of Biological Sciences and Environment Institute at The University of Adelaide, in Adelaide, South Australia, in Australia. Heidi K. Alleway and Robert C. Jones are affiliated with Provide Food and Water Sustainably, at The Nature Conservancy, in Arlington, Virginia, in the United States. Seth J. Theuerkauf is affiliated with the Office of Aquaculture at NOAA National Marine Fisheries Service, in Silver Spring, Maryland, in the United States.
